# Progress in the Circular Arc Source Structure and Magnetic Field Arc Control Technology for Arc Ion Plating

**DOI:** 10.3390/ma18153498

**Published:** 2025-07-25

**Authors:** Hao Du, Ke Zhang, Debin Liu, Wenchang Lang

**Affiliations:** 1 School of Naval Architecture and Ocean Engineering, Guangzhou Maritime University, Guangzhou 510725, China; duhao@gzmtu.edu.cn; 2 College of Mechanical and Electrical Engineering, Nanjing University of Aeronautics and Astronautics, Nanjing 210016, China; zhangkenh@nuaa.edu.cn; 3 Guangdong Jinhong New Materials Co., Ltd., Foshan 528200, China; jh_cip001@126.com; 4 Key Laboratory of Surface Modification, School of Intelligent Manufacturing, Wenzhou Polytechnic, Wenzhou 325035, China

**Keywords:** arc ion plating, circular arc source, magnetic field configuration, arc spot motion, macroparticles

## Abstract

Aiming at the goal of preparing high-quality coatings, this paper reviews the progress on circular arc source structure and magnetic field arc controlling technology in arc ion plating (AIP), with a focus on design characteristics of the different structures and configuration optimization of the corresponding magnetic fields. The circular arc source, due to its simple structure, convenient installation, flexible target combination, high cooling efficiency, and high ionization rate and deposition rate, has shown significant application potential in AIP technology. In terms of magnetic field arc controlling technology, this paper delves into the design progress of various magnetic field configurations, including fixed magnetic fields generated by permanent magnets, dynamic rotating magnetic fields, axially symmetric magnetic fields, rotating transverse magnetic fields, and multi-mode alternating electromagnetic coupling fields. By designing the magnetic field distribution reasonably, the trajectory and velocity of the arc spot can be controlled precisely, thus reducing the generation of macroparticles, improving target utilization, and enhancing coating uniformity. In particular, the introduction of multi-mode magnetic field coupling technology has broken through the limitations of traditional single magnetic field structures, achieving comprehensive optimization of arc spot motion and plasma transport. Hopefully, these research advances provide an important theoretical basis and technical support for the application of AIP technology in the preparation for high-quality decorative and functional coatings.

## 1. Introduction

In the vast landscape of thin film preparation technologies, a diverse array of methods has emerged to meet the ever-evolving demands of various industries. These technologies can be broadly categorized into physical vapor deposition (PVD), chemical vapor deposition (CVD), and other emerging techniques. Physical vapor deposition (PVD) includes magnetron sputtering (MS) and arc ion plating (AIP) as key methods. Compared to CVD, which relies on chemical reactions in the gas phase to form thin films, PVD techniques generally operate at lower temperatures, which is a crucial advantage when dealing with heat-sensitive substrates such as polymers and certain high-alloy materials. This lower temperature operation reduces the risk of thermal damage to the substrate, expanding the range of applicable materials. However, CVD often excels in achieving superior step coverage, making it more suitable for complex microelectronic structures with intricate trenches and vias [1,2,3].

Within the realm of PVD, magnetron sputtering has gained widespread use due to its ability to produce films with relatively low surface roughness and fewer macroparticles. This makes it a preferred choice in applications where surface smoothness is of utmost importance, such as in precision optical coatings. However, when compared to arc ion plating, magnetron sputtering falls short in several key aspects. The high ionization characteristics of AIP in physical vapor deposition bring about significant advantages. It leads to a marked improvement in film density when depositing hard coatings, effectively controlling porosity at an extremely low level. This, in turn, greatly enhances the wear resistance and corrosion resistance of the coatings. Moreover, the high ionization of AIP results in an outstanding film-substrate bonding strength, rendering it more suitable for high-load applications like those in the aerospace industry [4,5].

When further comparing AIP with other PVD techniques such as direct current (DC) sputtering and alternating current (AC) sputtering, the advantages of AIP become even more pronounced. Compared to DC sputtering, the arc discharge mechanism of AIP endows it with higher deposition efficiency. This is particularly evident when processing high-melting-point metal targets, where AIP can obtain well-crystallized films without the need for additional substrate heating. DC sputtering, on the other hand, with its stable discharge and controllable but relatively low deposition rate, is more favored in scenarios requiring ultra-thin film deposition with high thickness precision, such as in microelectronics for 10–50 nm barrier layers. AC sputtering has the ability to reduce target poisoning, which is a common issue in some sputtering processes. However, when it comes to preparing multi-element metal compound coatings, AIP shows significantly better control over composition uniformity through multi-target collaborative arc deposition. This superior control over composition is crucial in ensuring the performance consistency of the coatings, especially in advanced applications where precise material properties are required [6,7,8,9,10].

In addition to these comparisons, AIP demonstrates unique advantages in specific fields. In the realm of decorative coatings, its high ionization rate contributes to better color uniformity and stronger weather resistance, making the coated products more visually appealing and durable in various environmental conditions. For the surface modification of polymer films, the high-energy ions generated by AIP can achieve nanoscale roughening of the material surface, with a modification depth far exceeding that of traditional sputtering technology. This unique property finds valuable applications in fields such as medical catheters and optical films, where surface properties play a vital role in device performance [11,12,13,14].

Despite its numerous advantages, arc ion plating is not without limitations. The most notable drawback is the generation of macroparticles during the deposition process using traditional arc sources. These macroparticles lead to an increase in coating surface roughness, which restricts its application in precision fields such as semiconductors and optical components. This limitation highlights the need for further research to address the macroparticle issue [15,16,17,18,19,20,21,22,23,24].

The arc source, as a key component of AIP equipment, directly affects both the quality and uniformity of the deposited coating. The cathode arc source typically serves as not only an evaporation source but also an ionization source, and its structure in the vacuum chamber has been designed diversely, including circular, conical, rectangular planar, and cylindrical (Table 1). Among these arc sources, conical arc sources show complex structures, high production costs, and issues with magnetic field interference, resulting in poor arc control and low cooling efficiency. Rectangular planar large arc sources have a limitation on their targets, making it difficult to prepare multi-component coatings, and also exhibit low target utilization rates, high coating costs, and potential V-shaped grooves on the target surface after long-term use, further affecting target utilization. Cylindrical arc sources have limited installation positions, low coating efficiency, poor flexibility in the deposition process, and a small discharge area, and require complex motion devices [25,26,27,28,29,30,31,32,33,34].

In recent years, with in-depth research on AIP technology, the unique advantages of the circular arc source have gradually emerged. This type of arc source has a simple structure and is easy to install, allowing flexible combinations of multiple targets for the preparation of multi-component coatings. The unique geometric design not only achieves more uniform target etching but also forms a more stable plasma distribution, reducing the generation of macroparticles effectively [35,36,37,38]. Magnetic field arc control technology, as a key means to optimize AIP process, can precisely control the trajectory and the velocity of the arc spot by designing the magnetic field distribution, further improving the quality and the uniformity of the coating and enhancing the controllability of the deposition process. Moreover, the circular arc source exhibits high ionization and deposition rates during the coating process, enabling the preparation of dense, uniform, and fine-grained coatings that significantly enhance coating performance [39]. Additionally, the circular arc source has high operational stability, uniform arc combustion, and low failure rates, and is suitable for long-term stable operation, giving it a significant advantage in large-scale industrial production. Its well-designed cooling system can reduce target temperature effectively, further improving coating quality [40]. These features make the circular arc source stand out in AIP technology and an ideal choice for preparing high-quality decorative and functional coatings.

Currently, extensive studies have been conducted on the structure of circular arc sources and their magnetic field arc control technology, achieving a series of important progress [25,26,27,41,42,43,44,45,46,47,48,49,50]. Our team has also been engaged in research and development on circular arc sources for twenty years [51,52]. It is the purpose of this paper to review design principles, optimization methods, and current application status of circular arc sources in AIP. Combining our experience and the available results, we will focus on analyzing and discussing the latest progress in magnetic field arc control technology, including composite magnetic field design, dynamic magnetic field application, and the effects of controlling magnetic field parameter on structure and properties of the deposited coatings.

## 2. Structural Features of Circular Arc Sources

The core component of a circular arc source is the circular planar cathode target, with a design diameter typically ranging from 60 mm to 160 mm and a thickness of about 30 mm to 40 mm. The target adopts a simple and efficient circular planar structure, which not only facilitates processing and installation but also provides a large evaporation area, thereby significantly enhancing the deposition rate of the coating. Behind the target, permanent magnets or electromagnetic coils are installed primarily to generate a magnetic field for arc control. Through the constraining effect of the magnetic field, the arc spots can move in an orderly way on the surface of the target, effectively avoiding random motion of the arc spots and thereby improving the stability of arc discharge [53,54].

The structural design of the circular arc source is highly flexible. The target can be flexibly combined with various materials according to different applications, making the preparation of multi-component coatings more convenient. The cooling system is usually installed on the back of the target, with coolant flowing through the cooling channels on the back of the target to remove the heat generated during the coating process effectively. This efficient cooling design not only helps maintain the temperature stability of the target but also further reduces the generation of macroparticles, thereby improving the quality of the coating significantly. Additionally, a shielding cover is always designed around the target to restrict the motion of the cathode spot and prevent it from transferring to non-evaporation surfaces, further ensuring the stability of the coating process [55]. By optimizing the target size, magnetic field layout, and cooling system, the structural design of the circular arc source achieves efficient and stable coating preparation. Its simple design, flexible material combination, and efficient cooling effect make it an ideal evaporation source structure in AIP technology.

The ability to design the magnetic field widely for the circular arc source is one of its core advantages. By optimizing the magnetic field distribution, the trajectory and the speed of the arc spot can be effectively controlled. The arc control magnetic field can generally be orthogonally decomposed into radial and axial magnetic fields. Among them, the radial magnetic field plays a decisive role in improving arc spot discharge, increasing the motion speed of the arc spot, and reducing the ejection of macroparticles [39,56].

**Figure 1:**
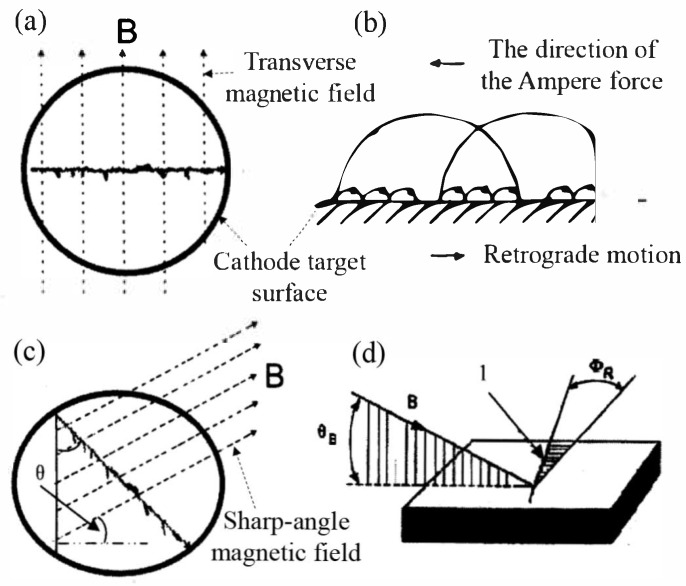
Schematic diagram of the effects of different magnetic field components on arc spot motion: (**a**) Transverse magnetic field parallel to the cathode target surface; (**b**) Influence of the transverse magnetic field on the motion of the arc spot (Retrograde motion); (**c**) Sharp-angle magnetic field intersecting the cathode surface at a certain angle; (**d**) Influence of the sharp-angle magnetic field on the motion of the arc spot (Acute angle principle).

When applying a radial magnetic field to the cathode target surface, the cathode arc spot will move in the direction opposite to the Ampère force, as shown in Figure 1a, and its motion speed shows a parabolic variation with the transverse magnetic field strength (Figure 1b), and this characteristic can effectively enhance the motion speed of the arc spot [56,57,58]. If an angle θ appears between the magnetic field and the cathode surface, i.e., forming an acute magnetic field with a magnetic induction intensity of B, the cathode arc spot will not only move in the opposite direction but also exhibit the Robson drift phenomenon (Figure 1c) [59,60]. According to the acute angle principle, the drift direction points to the acute angle region θ_B_ between the magnetic field lines and the cathode target surface. In Figure 1d, the angle Φ_R_ between the motion direction of the arc spot and the intersection line of the magnetic field lines with the target surface is approximately equal to θ_B_. Using the acute angle principle, the magnetic field can constrain the trajectory of the arc spot and precisely control its position on the target surface. This is of key significance designing and tailoring the magnetic field configurations that optimize the trajectory of the arc spot and ensure uniform target etching [61].

Actually, all magnetic field designs mentioned above are based on forming specific magnetic field configurations on the target surface [62,63,64,65]. By utilizing the acute angle principle to restrict the motion of the arc spot and leveraging the transverse magnetic field component to increase the speed of the arc spot, a ring-shaped rotating arc spot around the center of the target is formed. Specifically, the magnetic field generated by permanent magnets forms an arch-shaped magnetic field configuration on the target surface. This arch-shaped magnetic field creates a closed path on the target surface, and the arc spot can perform circumferential rotational motion on the target surface through the constraining effect of the magnetic field. This design not only constrains the motion of the arc spot effectively, preventing it from concentrating in local areas that cause localized overheating and uneven target erosion, but also increases the speed of the arc spot and reduces the generation of macroparticles. However, the fixed design of the magnetic field distribution configuration largely limits the utilization rate of the target and can also lead to an increased ejection of macroparticles, thereby affecting coating quality [66,67].

To achieve uniform etching of the whole target surface, the current approach typically involves introducing a composite magnetic field that combines electromagnetic coils with permanent magnets. This design not only provides basic magnetic field confinement but also allows for dynamic adjustment of the magnetic field strength and configuration by regulating the current in the electromagnetic coils, thereby further optimizing the trajectory and the speed distribution of the arc spot. On one hand, by applying dynamic or scanning magnetic fields, the area and intensity of the transverse magnetic field component are maximized to enable the arc spot to move orderly over a wider area, thus improving the overall utilization rate of the target [68,69,70,71,72]. On the other hand, by designing specific magnetic field configurations to control the trajectory of the arc spot precisely, it can cover the entire target surface uniformly, further reducing the issues of localized overheating and uneven erosion [63].

In the magnetic field configuration design for new-type arc sources, all different magnetic field configurations adopt a circular arc source structure with multiple sets of composite permanent magnets or permanent magnet–electromagnetic coil combinations. By adjusting the electromagnetic field strength, this design can form a coupled magnetic field to regulate the strength of the radial and axial magnetic fields on the cathode target surface. This flexible capability on controlling magnetic field not only improves the etching condition of the target, making it more uniform, but also optimizes the emission state of the plasma, thereby enhancing the quality and performance of the deposited coatings. Through this composite magnetic field design, some of the limitations of traditional fixed magnetic field designs can be overcome, providing a new idea and direction for the development of AIP technology.

## 3. Magnetic Field Configuration Design

### 3.1. Permanent Magnet Rotating Magnetic Field Arc Source

**Figure 2:**
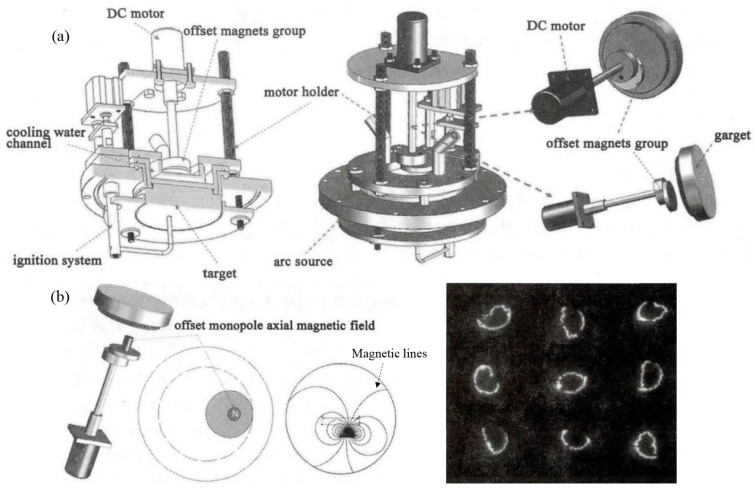

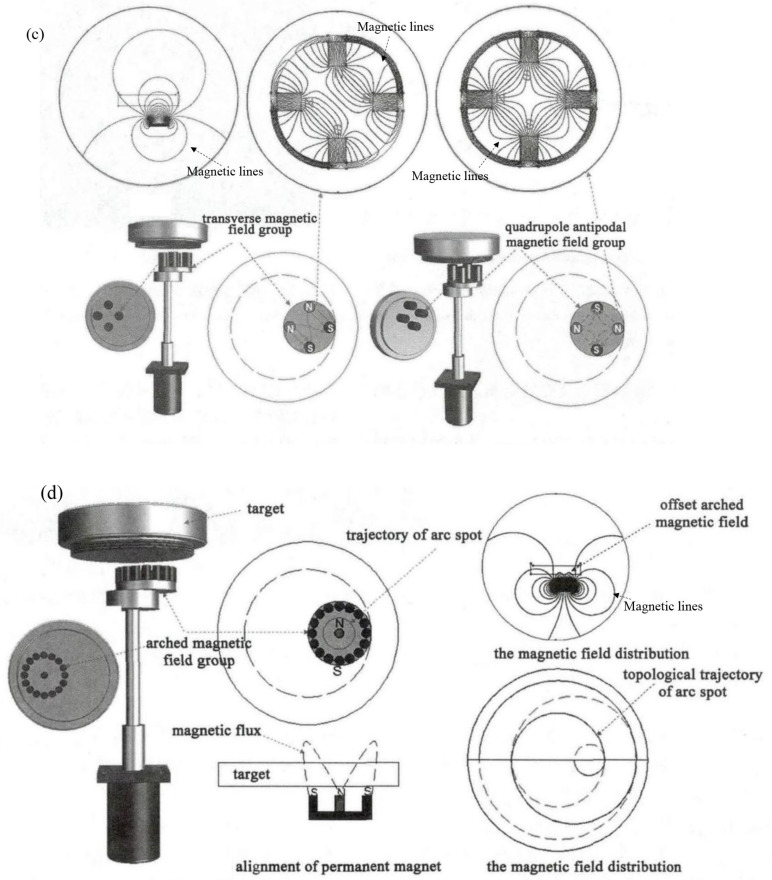
Schematic diagram of the multi-mode permanent magnet rotating magnetic field arc source and its magnetic field configuration: (**a**) Schematic diagram of a mechanically rotating magnetic field arc source; (**b**) Eccentric single-pole axial magnetic field device, magnetic field configuration, and corresponding arc spot morphology; (**c**) Schematic diagram of the eccentric transverse magnetic field device and eccentric quadrupole opposite magnetic field device, as well as their magnetic field configurations; (**d**) Schematic diagram of the eccentric arch-shaped magnetic field device and its magnetic field configuration.

Considering the randomness of arc motion in traditional arc deposition, permanent magnets are used to generate an arch-shaped magnetic field. This design guides the arc to move along a preset trajectory, allowing circular motion on the cathode surface. Although this design achieves preliminary control of arc motion to some extent, it also has obvious limitations. Since the magnetic field configuration generated by permanent magnets is fixed, the motion area of the arc spot is restricted, resulting in low target utilization. Additionally, the high temperature in the arc spot motion area not only affects the quality of the deposited coatings but also may cause local overheating and uneven erosion of the target. To overcome the limitations of traditional permanent magnet magnetic fields, permanent magnet rotating magnetic field arc sources have been designed [69,70]. This type of arc source introduces a rotatable magnetic field that can adjust the motion area of the arc spot dynamically. This dynamic adjustment capability enables the magnetic field to be optimized in real-time as needed, improving the uniformity and the controllability of arc motion significantly, as shown in Figure 2a.

The design of the rotating magnetic field device based on permanent magnets is diverse. For example, the eccentric single-pole axial magnetic field (as shown in Figure 2b) is designed by placing a single permanent magnet at a position one-quarter of the target diameter on the magnetic yoke disk, forming an asymmetric axial magnetic field. The magnetic field forms an asymmetric axial distribution on the target surface, with the longitudinal component of the magnetic field being the strongest at the center of the permanent magnet and the transverse component gradually increasing towards both sides. The arc spot forms a “semi-circular” trajectory at the edge of the target and exhibits rotational motion. The arc spot also expands in the strong magnetic field area, covering the entire target surface. This design increases the motion speed of the arc spot, expands the discharge area, reduces the discharge power density, and decreases the ejection of macroparticles, thereby improving the utilization rate of the target significantly.

The eccentric transverse magnetic field is formed by placing four single-pole permanent magnets at right-angle positions around the magnetic yoke disk, creating a closed transverse magnetic field, as shown in Figure 2c. The magnetic field forms a closed transverse distribution on the target surface, with the transverse component of the magnetic field being the strongest and the longitudinal component being zero. Under the influence of the magnetic field, the arc spot moves in a straight line in a fixed direction, with its trajectory covering a large part of the target surface [69]. The linear motion of the arc spot helps to achieve uniform discharge, further improving the quality and the uniformity of the deposited coatings.

The eccentric arch-shaped magnetic field (as shown in Figure 2d) is formed by placing magnetic steel with opposite polarities at the center and periphery on the magnetic yoke disk, creating an arch-shaped magnetic field. The magnetic field forms an eccentric arch-shaped distribution on the target surface, divided into a closed weak arch-shaped magnetic field area and a strong diffusion magnetic field area. The arc spot forms an approximately circular trajectory under the constraint of a weak arch-shaped magnetic field [69,70]. As the magnetic field dynamically changes, the arc spot enters the strong diffusion magnetic field area and exhibits a spiral trajectory, which covers the entire target surface.

Through these innovative magnetic field designs, the permanent magnet rotating magnetic field arc source not only overcomes the limitations of traditional permanent magnet magnetic field designs but also achieves a precise control of arc spot motion, improving the utilization rate of the target and the quality of the deposited coatings significantly. These advancements provide new ideas and directions for the development of AIP technology.

### 3.2. Axially Symmetric Magnetic Field Arc Source

**Figure 3:**
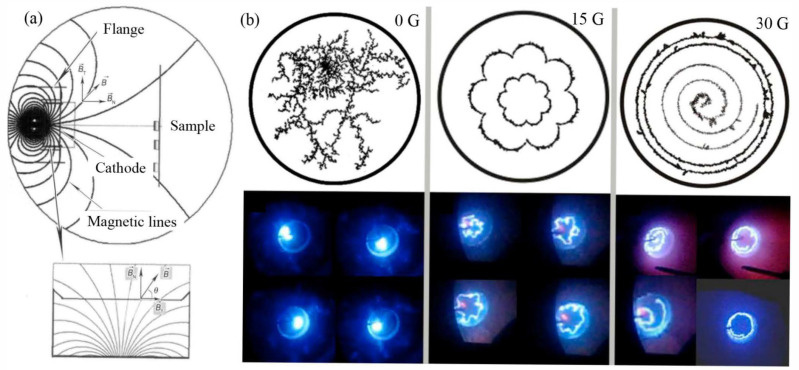
Axially symmetric magnetic field configuration and arc spot motion trajectories: (**a**) Axially symmetric magnetic field; (**b**) Trajectories of arc spot motion under different magnetic field strengths.

The design of an axially symmetric magnetic field typically employs an adjustable current electromagnet coil installed behind the target to generate the magnetic field. To enhance the strength and uniformity of the magnetic field, a high-permeability nickel-plated pure iron material is always placed at the center of the coil. This design results in an axially symmetric distribution of the magnetic field on the target surface, as shown in Figure 3a, indicating that the strength and direction of the magnetic field are symmetric about the central axis of the target. Specifically, the transverse magnetic field strength is the highest at the edge of the target and zero at the center. By adjusting the current in the coil, the strength of the magnetic field can be controlled flexibly, thereby achieving precise regulation of the arc spot motion. The axially symmetric magnetic field influences the distribution of spatial positive charge density, thereby altering the electric field strength and the work function of the cathode material, which affects the formation position of new arc spots significantly.

When there is no external magnetic field or the magnetic field strength is weak (B_t_ < 5 G), the arc spot exhibits random and irregular motion on the target surface, as shown in Figure 3b. This is because, in the absence of an external magnetic field, the formation position of the arc spot is entirely determined by local instability factors, such as the roughness of the target surface and the impact force of metal vapor. These factors result in irregular motion of the arc spot, making it difficult to achieve an effective control on the motion.

However, in the cases when the strength of the axially symmetric magnetic field gradually increases to some extent (5 G < B_t_ < 18 G), the behavior of the arc spot motion begins to change significantly. The arc spot starts to exhibit a rotational motion trend and expands towards the edge of the target. The motion speed of the arc spot increases, and its trajectory gradually shifts from random motion to regular, rotationally controlled one.

When the strength of the axially symmetric magnetic field increases further and reaches a critical value (B_t_ ≈ 30 G), the arc spot stably and rapidly rotates at the edge of the target, with an up-and-down jitter along the target edge. At this point, the arc spot splits into multiple fine circular spot lines, and several fine arc spot lines appear at the center of the target approximately every 0.5 s, then quickly spiral outward and disappear. This phenomenon indicates that under the influence of a strong magnetic field, the motion of the arc spot becomes more orderly and uniform, achieving comprehensive coverage of the target surface, thereby significantly improving the utilization rate of the target and the uniformity of the deposited coatings [73,74].

### 3.3. Rotating Transverse Magnetic Field Arc Source

The arc spot is primarily driven by the Lorentz force in the direction opposite to the Ampère force under the influence of a transverse magnetic field. To achieve a precise control over the trajectory of the arc spot and to break through the traditional static or quasi-static magnetic field designs as well as mechanically dynamic magnetic field designs, researchers have developed a rotating magnetic field arc source with adjustable speed and amplitude for AIP to control the arc spot motion [75,76].

**Figure 4:**
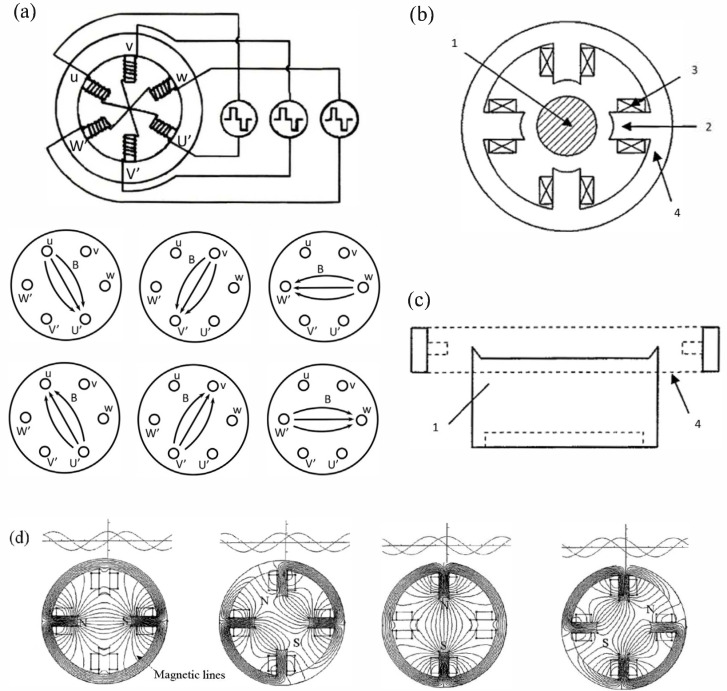
Schematic diagram of the rotating transverse magnetic field arc source and magnetic field configurations: (**a**) Schematic diagram of the principle of the rotating transverse magnetic field; (**b**) Schematic diagram of rotating magnetic field generator. 1. Target; 2. Magnetic pole; 3. Electromagnetic coil; 4. Generator; (**c**) Schematic diagram of the position between rotating magnetic field generator and target; (**d**) Simulated diagrams of the transient magnetic field distribution of the rotating magnetic field generator at different instants within one current cycle.

As shown in Figure 4, the design of the rotating magnetic field arc source for AIP involves placing a rotating magnetic field generation device around the target. This device consists of multiple magnetic poles distributed along the same circumference evenly, forming an electromagnetic circuit framework. The excitation coils are either wrapped around the magnetic poles or embedded in the gaps between adjacent magnetic poles. By using a two-phase excitation sequence with a phase difference of 90° or a three-phase excitation sequence with a phase difference of 120°, a controllable rotating magnetic field is generated. This design not only enables dynamic changes in the magnetic field but also allows precise control of the rotation speed and strength of the magnetic field by adjusting the frequency and magnitude of the excitation current.

The rotating magnetic field generation device can be placed either inside or outside the vacuum chamber, with adjustable positioning, and the effective region of the magnetic field should encircle the target surface [61]. Furthermore, by using a PLC controller and a frequency converter, the frequency and magnitude of the excitation current can be precisely regulated, thereby achieving more precise control over the motion of the arc spot and the discharge pattern. This design reduces the ejection of macroparticles effectively, enables distributed discharge, and improves the quality and the uniformity of the deposited coatings significantly [77].

As the strength and the frequency of the rotating transverse magnetic field increase, the arc spot transits from a clustered state under low transverse magnetic field strength to widely distributed arc spot lines covering the target surface, and finally to strongly dispersed arc spot lines that cover the entire target surface completely. This transition indicates that the rotating magnetic field can change the motion state and discharge pattern of the arc spot effectively.

The rotating transverse magnetic field alters the trajectory and the distribution of the charged macroparticles, affecting the spatial charge layer density, thereby changing the motion state and discharge pattern of the arc spot. Specifically, the dynamic nature of the rotating magnetic field allows the arc spot to move more uniformly and orderly on the target surface, avoiding excessive concentration of the arc spot in localized areas, which reduces the generation of macroparticles. Moreover, the adjustability of the strength and the frequency of the rotating magnetic field enables a precise control over the trajectory and the speed of the arc spot, further enhancing the quality and the uniformity of the deposited coatings [51,71,75,76,77].

### 3.4. Multi-Mode Alternating Electromagnetic Coupled Arc Source

**Figure 5:**
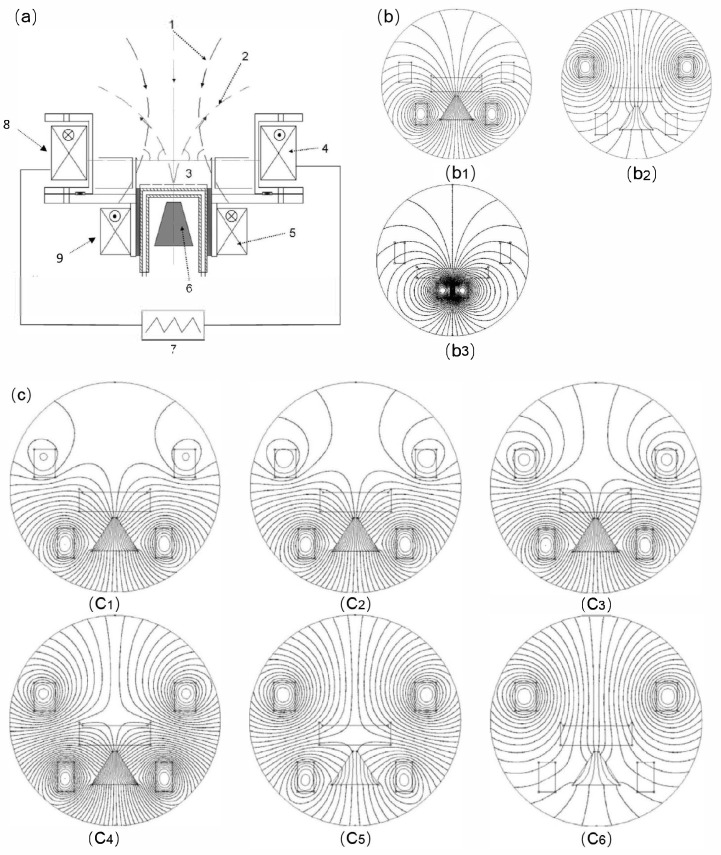
Schematic diagram of the multi-mode alternating coupled arc source and magnetic field configurations: (**a**) Schematic diagram of the efficient dynamic coupled magnetic field arc source device formed by the superposition of a static axially symmetric divergent magnetic field and a reverse dynamic focusing guiding magnetic field. 1. Focusing guiding magnetic field; 2. Axially symmetric divergent magnetic field; 3. Small-diameter cylindrical target; 4. Focusing guiding electromagnetic coil; 5. Axially symmetric electromagnetic coil; 6. Magnetic yoke; 7. Variable frequency power supply; 8. Focusing guiding magnetic field generation device; 9. Axially symmetric divergent magnetic field generation device; (**b**) Schematic diagram of different magnetic field configurations on the target surface: (**b_1_**) Axially symmetric divergent magnetic field pointing towards the edge of the target; (**b_2_**) Axial focusing guiding magnetic field pointing towards the center of the target; (**b_3_**) Axially symmetric arch-shaped magnetic field; (**c**) Schematic diagram of the variation in coupled magnetic field lines due to an increase in the strength of the reverse focusing guiding magnetic field while the axially symmetric divergent magnetic field strength remains constant: (**C_1_**–**C_6_**) correspond to the gradual increase in the strength of the reverse focusing guiding magnetic field.

The introduction of multi-mode alternating coupled magnetic fields provides a new approach for optimizing arc spot motion and improving coating quality [78]. As shown in Figure 5a, the dynamic coupled magnetic field generation device mainly consists of an axially symmetric magnetic field generation device and a focusing guiding magnetic field generation device. The axially symmetric magnetic field generation device is placed behind the target and composed of a high-permeability magnetic yoke and an electromagnetic coil coaxially positioned with the yoke, or it can be formed by a single or multiple permanent magnets in combination with the yoke. The focusing guiding magnetic field generation device is placed in front of the target, coaxially aligned with the target, and the center of the device is flush with or slightly higher than the target surface.

The axially symmetric magnetic field generation device produces an axially symmetric divergent magnetic field, forming an acute angle pointing towards the edge of the target, which can push the arc spot outward, as shown in Figure 5(b_1_). Meanwhile, the focusing magnetic field above the target surface forms an opposite acute angle pointing towards the center of the target, as shown in Figure 5(b_2_), which can confine the arc spot to the center of the target. The dynamic superposition of these two tendencies allows for a dynamic control of the arc spot motion. For example, a static axially symmetric divergent magnetic field can be combined with a dynamic focusing guiding magnetic field, or a dynamic axially symmetric divergent magnetic field can be combined with a static focusing guiding magnetic field. The axially symmetric magnetic field can also be compounded to form an axially symmetric arch-shaped magnetic field, as shown in Figure 5(b_3_), to further dynamically control the arc spot motion area precisely. For instance, a static axially symmetric arch-shaped magnetic field can be coupled with a dynamic focusing axial guiding magnetic field, or an axially symmetric arch-shaped magnetic field with periodically varying intensity can be coupled with a focusing axial guiding magnetic field of constant strength.

Figure 5c illustrates the magnetic field configuration changes of an efficient dynamic coupled magnetic field arc source device formed by the superposition of a static axially symmetric divergent magnetic field and a reverse dynamic focusing guiding magnetic field. This reverse focusing guiding magnetic field varies from small to large in the form of a triangular wave or half-sine wave periodically. The dynamic superposition of these two tendencies can control the arc spot motion and improve the arc spot discharge state dynamically.

**Figure 6:**
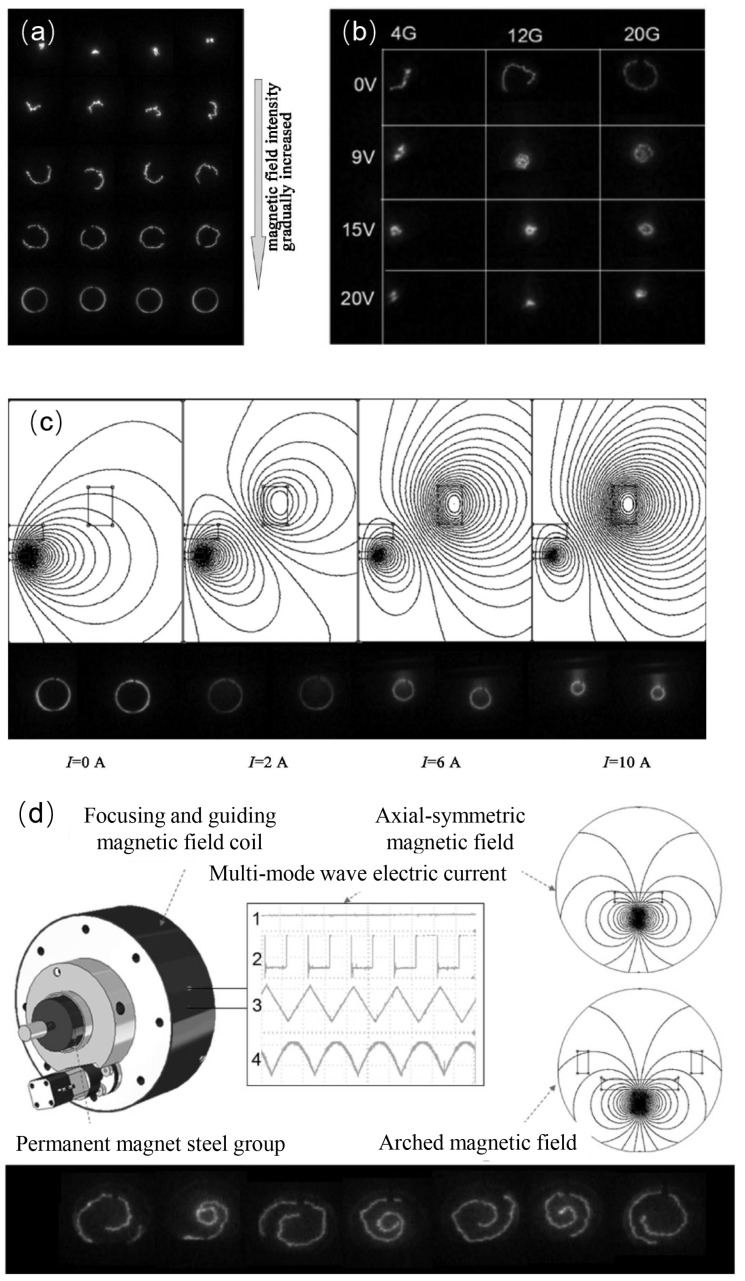
Influence of multi-mode alternating coupled magnetic field configurations on arc spot motion: (**a**) Influence of the axially symmetric divergent magnetic field configuration on arc spot motion; (**b**) Influence of the axially symmetric focusing magnetic field configuration on arc spot motion; (**c**) Influence of the static axially symmetric arch-shaped magnetic field configuration on arc spot motion; (**d**) Schematic diagram of the multi-mode alternating coupled magnetic field-assisted arc ion plating arc source device and the discharge morphology of the arc spot under 15 Hz sine wave mode.

As shown in Figure 6a, the axisymmetric divergent magnetic field tends to push the arc spot to expand outward. As the magnetic field strength increases, the arc spot gradually drifts toward the edge of the target and superimposes a circumferential reverse rotational motion. The magnetic field strength can be controlled by adjusting the current magnitude or the number of coil turns of the magnetic field generating device, so as to adjust the trajectory of the arc spot. The reverse-polarity focusing guiding magnetic field, with the increasing voltage in the electromagnetic coil, restrains the arc spot back to the center of the target surface, reducing plasma divergence, as shown in Figure 6b. The axially symmetric arch-shaped magnetic field confines the arc spot to a fixed trajectory. When the multi-mode reverse-polarity dynamic focusing guiding magnetic field is superimposed with the axially symmetric divergent or arch-shaped magnetic field, a dynamic arch-shaped coupled magnetic field is formed. This dynamic coupled magnetic field can flexibly control the motion and improve the discharge state of the arc spot, thereby reduce macroparticles ejection, as shown in Figure 6c.

Under the guidance of the focusing magnetic field, the plasma can be stably transmitted, while increasing the collision probability of macroparticles in the plasma, resulting in an enhancement in both ionization rate and ion density. The reverse-polarity dynamic focusing guiding magnetic field coil is driven by a multi-waveform electromagnetic coil control power supply, which can output direct current (DC) and DC-biased alternating current (AC) in the form of triangle waves, rectangular waves, bell-shaped waves, sine waves, and other forms of alternating current, with independently adjustable frequency and amplitude. This enables a multi-mode control of the arc spot, as shown in Figure 6d. This design not only enhances the flexibility of the coating process but also provides an effective technical means to address the issue of macroparticles [70,71].

### 3.5. Multi-Magnetic Field Structure Coupled Arc Source

#### 3.5.1. Multi-Level Magnetic Field Coupling

Traditional single-magnetic field configurations, although capable of guiding the motion of the arc spot on the target surface, often result in limited trajectories. Moreover, single-magnetic field configurations also have certain limitations in controlling the motion speed of the arc spot and the efficiency of plasma transport, which restricts further improvements in quality and deposition efficiency of the deposited coatings. To overcome these limitations, multi-level magnetic field coupling technology was introduced [79].

Multi-level magnetic field coupling technology introduces multiple magnetic field levels with different gradients, designing multiple magnetic field regions around the target, which can significantly and multi-level adjustably change the motion pattern of the arc spot. Specifically, multi-level magnetic field coupling can not only achieve rapid rotation and uniform distribution of the arc spot by adjusting the strength and the direction of the magnetic field but also optimize the trajectory of the arc spot and the efficiency of plasma transport through dynamic adjustment of the magnetic field in gradient and distribution. This technology can also reduce the ejection of macroparticles by optimizing the magnetic field distribution, thereby improving the adhesion and the surface quality of the deposited coatings. The introduction of multi-level magnetic field coupling technology not only enhances the flexibility of the AIP process but also provides new technical means for achieving an efficient and uniform coating deposition.

**Figure 7:**
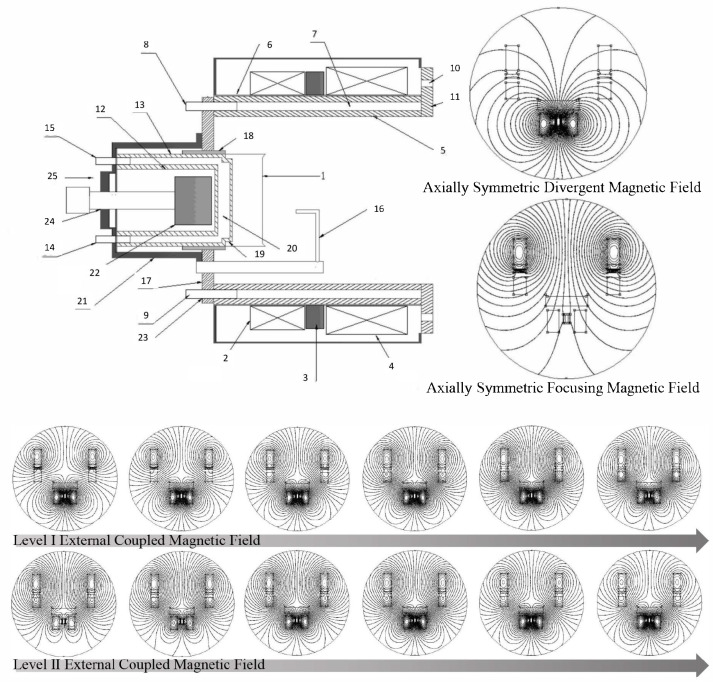
Multi-level coupled axially symmetric magnetic field-constrained AIP device. 1. Target; 2. Level I outer coupled magnetic field generation device; 3. Magnetic yoke; 4. Level II outer coupled magnetic field generation device; 5. Flange sleeve; 6. Flange sleeve insulator; 7. Flange sleeve cooling water channel; 8. Flange sleeve outlet; 9. Flange sleeve inlet; 10. Flange installation hole; 11. Flange plate; 12. Inner cylinder; 13. Outer cylinder; 14. Target base outlet pipe; 15. Target base inlet pipe; 16. Arc ignition device; 17. Target base baseplate; 18. Target base insulator; 19. Connecting thread; 20. Target base cooling water channel; 21. Target base shield; 22. Permanent magnet device; 23. Target base baseplate connection hole; 24. Permanent magnet device installation hole; 25. Target base.

Figure 7 is a schematic diagram of the non-equilibrium dynamic arch-shaped compatible axial guiding magnetic field-assisted device and the variation of the magnetic field configuration. The device in the figure consists of an inner coupled magnetic field generation device and two sets of outer coupled magnetic field generation devices, forming a multi-level composite magnetic field that is compatible with the dynamic arch-shaped magnetic field on the target surface for constraining arc spot motion and the axial focusing guiding magnetic field in the transport space.

Generally, the inner coupled magnetic field generation device is placed behind the target and is mainly composed of a permanent magnet device or an inner coupled magnetic field generation device with an iron core set at the center of the coil. This design can produce an axially symmetric inner coupled magnetic field, providing a stable background magnetic field for the initial motion of the arc spot. The outer coupled magnetic field generation device is placed in front of the target and is coaxially set with the target. Among them, the Level I outer coupled magnetic field generation device is used in conjunction with the inner coupled magnetic field generation device to form the dynamic arch-shaped magnetic field on the target surface. The Level II outer coupled magnetic field generation device is used in conjunction with the Level I outer coupled magnetic field generation device to form the axial focusing guiding magnetic field in the transport space.

The strength and the direction of the dynamic arch-shaped magnetic field on the target surface can be dynamically adjusted to form a dynamic arch-shaped magnetic field with a strength in a range of 10–150 Gauss. Both the Level I and the Level II outer coupled magnetic fields are axial ones. The strength of the Level II outer coupled magnetic field remains constant and is generated by an electromagnetic coil powered by direct current, forming a focusing guiding magnetic field with a strength between 100 Gauss and 250 Gauss. The electromagnetic coils in the inner coupled magnetic field generation device and the two sets of outer coupled magnetic field generation devices can be adjusted individually or collectively. When the electromagnetic coils are powered by a direct current, the magnetic field strength is controlled by the current intensity. When powered by alternating current, the form of the alternating current can be a frequency-adjustable direct current-biased triangular wave, sawtooth wave, pulse square wave, half-sine wave, sine wave, or other forms of alternating current. The amplitude of the alternating current voltage is adjustable, the same as that of the bias current voltage.

#### 3.5.2. Static and Dynamic Magnetic Field Coupling

The coupling of static and dynamic magnetic fields has been widely applied in the various magnetic field arc control designs mentioned above [80,81,82,83,84,85]. This design combines the stability of static magnetic fields with the flexibility of dynamic magnetic fields cleverly, achieving a precise control over the motion of the arc spot and the transport of plasma through carefully designed spatiotemporal distributions of the magnetic fields.

Specifically, the static magnetic field provides a stable background field for the arc spot, ensuring its stable operation on the target surface. The dynamic magnetic field, through periodic or non-periodic variations, further optimizes the trajectory and speed of the arc spot, enabling it to achieve a more uniform distribution and more efficient etching on the target surface. This coupling method not only reduces the excessive dwell duration of the arc spot in local areas effectively, avoiding local over-etching of the target, but also enhances the transport efficiency of the plasma by dynamically adjusting the strength and direction of the magnetic field, thereby improving the uniformity and adhesion of the film. Moreover, the coupling of static and dynamic magnetic fields can be flexibly adjusted according to different process requirements, achieving a precise control over the motion of arc spot and the plasma transport, thus playing an important role in various AIP application scenarios.

The combination of permanent magnets and electromagnetic coils for static and dynamic magnetic fields offers a considerable flexibility. Generally, this can be achieved by installing permanent magnets in a transverse (radial to the target) or longitudinal (perpendicular to the target) orientation on the cathode base and cathode cover, in conjunction with the magnetic field regulation of electromagnetic coils, to realize various magnetic field modes, such as the composite periodic electromagnetic mode with central transverse and peripheral longitudinal configurations, and the composite programmable electromagnetic mode with dual-longitudinal configurations at the center and the periphery.

**Figure 8:**
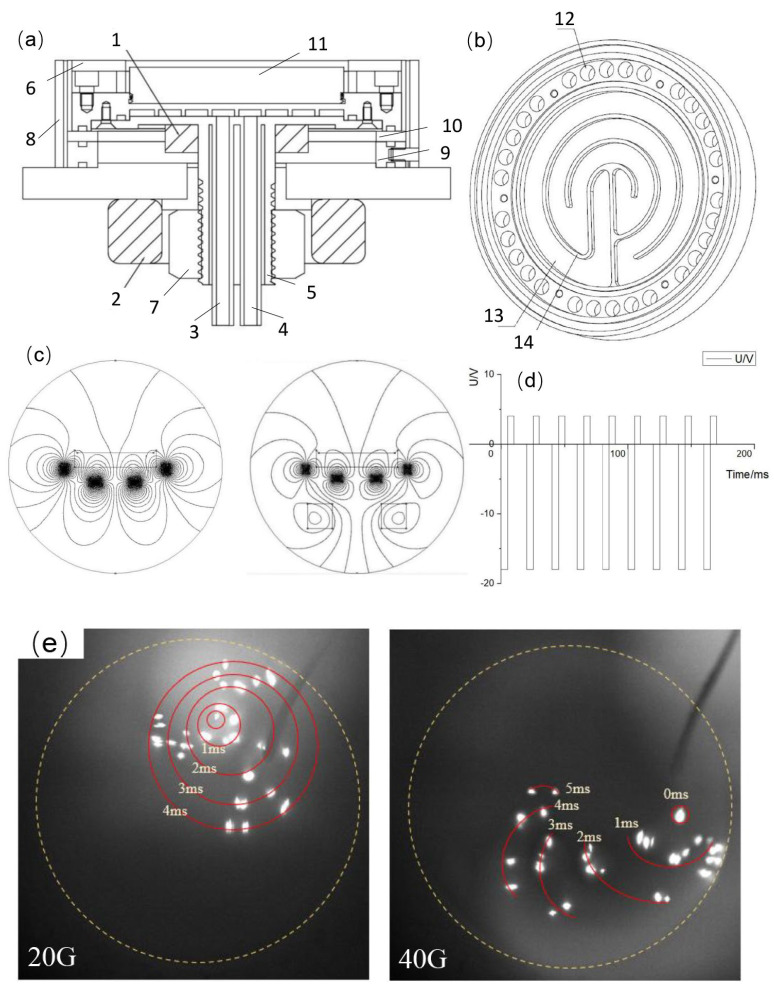
Schematic diagram of the arc source device with static and dynamic coupled magnetic fields. 1. Permanent magnet installation piece; 2. Electromagnetic coil; 3. Water inlet pipe; 4. Water outlet pipe; 5. Connecting sleeve; 6. Shielding pressure plate; 7. Compression nut; 8. Dust cover; 9. Sealing ring; 10. Insulating plate; 11. Target; 12. Permanent magnet installation hole; 13. Water-cooled inner cavity; 14. Partition. (**a**) Schematic diagram of the structure of the composite periodic electromagnetic mode with central transverse and peripheral longitudinal configurations; (**b**) Schematic diagram of the magnetic field structure at the edge of the cathode base; (**c**) Schematic diagram of the magnetic field simulation; (**d**) Schematic diagram of the coil current output; (**e**) Schematic diagram of high-current pulse arc splitting under different transverse magnetic field strengths.

As shown in Figure 8, the composite periodic electromagnetic mode with central transverse and peripheral longitudinal configurations involves equipping the central permanent magnet holder with transverse permanent magnets, whose magnetic field direction is aligned with the radial direction of the target, generating a strong horizontal magnetic field component. This design helps enhance the deflection ability of electrons in the horizontal direction, thereby promoting greater ionization of electrons and improving the ionization efficiency of the target significantly. Meanwhile, longitudinal permanent magnets are installed in the permanent magnet holes on the cathode base, with their magnetic field direction perpendicular to the target surface. These longitudinal permanent magnets serve to enhance the longitudinal magnetic field component in front of the target, thereby increasing the density of the plasma and enhancing its transport capability towards the workpiece.

On the basis of the central transverse and peripheral longitudinal permanent magnet combination, further integration of controllable multi-waveform alternating coil currents is implemented. By dynamically altering the magnetic field configuration through alternating current, the arc spot is induced to superimpose reciprocating radial motion on its circumferential motion, enhancing the utilization rate of the target significantly. Building on this foundation, the application of modulated pulsed arcs can instantaneously apply high-intensity currents to the target surface. On one hand, this high-intensity current increases the magnetic field strength on the target surface markedly, causing the arc spot to split and form the so-called “splitted arc” phenomenon [64,65,86,87,88,89,90,91,92]. This arc spot splitting helps reduce the local energy density and temperature on the target surface, thereby decreasing the generation of macroparticles and consequently improving the uniformity and the quality of the deposited coatings. On the other hand, the superposition effect of the instantaneous high current allows for a substantial reduction in the base current required to maintain arc stability. This not only further reduces the formation of macroparticles but also enhances the intensity of the plasma during the arc discharge process, increasing the ionization rate of the cathode and thereby improving the efficiency and the quality of the entire coating process significantly.

#### 3.5.3. Overview of Multi-Mode Magnetic Field Coupling

Axially symmetric magnetic fields can provide a stable background field, ensuring the uniform distribution of the arc spot on the target surface; transverse magnetic fields can guide the arc spot to move in a specific direction, reducing local over-etching; and rotating magnetic fields can further optimize the trajectory of the arc spot and the efficiency of plasma transport by dynamically adjusting the direction of the magnetic field. Multi-mode magnetic field coupling technology integrates various modes of magnetic fields and combines the characteristics of static and dynamic magnetic fields to achieve comprehensive optimization of arc spot motion and plasma transport. This coupling method not only guides the arc spot to form complex trajectories on the target surface, such as spiral and circular motions, through the synergistic action of different modes of magnetic fields, but also further optimizes the speed and the uniformity of arc spot motion by dynamically adjusting the strength, direction, and distribution of the magnetic field [83,93,94,95,96,97].

**Figure 9:**
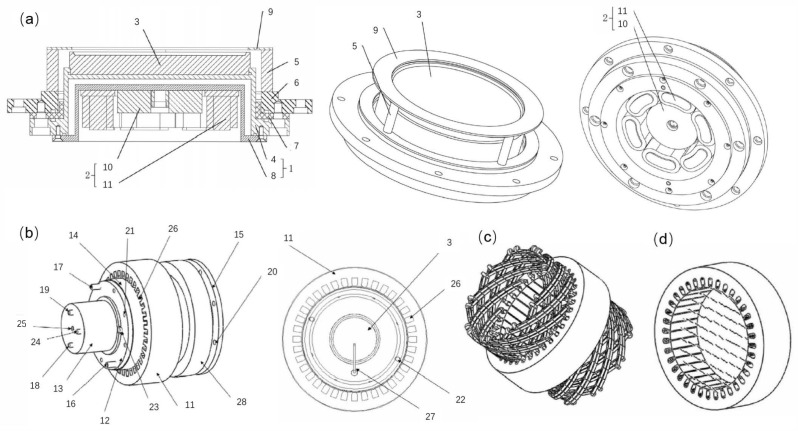
Multi-mode magnetic field coupled arc source device. 1. Cathode body; 2. Magnet; 3. Target; 4. Cathode base; 5. Shield support rod; 6. Base; 7. Insulator; 8. Cathode rear cover; 9. Shield ring; 10. Central magnetic group; 11. Secondary transverse rotating magnetic field generation device; 12. Target base baseplate; 13. Target base shield; 14. Flange sleeve insulator; 15. Flange plate; 16. Flange sleeve inlet; 17. Flange sleeve outlet; 18. Target base outlet; 19. Target base inlet; 20. Flange connection hole; 21. Target base baseplate connection hole; 22. Target base baseplate connection hole; 23. Arc ignition device installation hole; 24. Power supply connector; 25. Permanent magnet device installation hole; 26. Rotating magnetic field generation device slot; 27. Arc ignition device; 28. Axial focusing guiding magnetic field generation device. (**a**) Schematic diagram of the structure of the central magnetic group and secondary transverse rotating magnetic field coupled arc source; (**b**) Schematic diagram of the structure of the quasi-diffusion arc cold cathode arc source; (**c**) Three-dimensional structure and winding distribution diagram of the bipolar radial rotating magnetic field and axial focusing guiding magnetic field combination generation device; (**d**) Cross-sectional transient magnetic field distribution diagram of the bipolar radial rotating magnetic field.

For example, the combination of a central magnetic group and a secondary transverse rotating magnetic field generation device, as shown in Figure 9a, places the central magnetic group at the center of the cathode body, parallel to the axial or target surface direction of the cathode body. It can be a single strong magnet, a group of multiple magnets, or even a composite magnetic field formed by two directional magnet groups. The central magnetic group primarily provides a stable static magnetic field that constrains the motion of the arc spot, enabling it to discharge stably in a specific area on the target surface and enhancing the stability of arc discharge. The secondary transverse rotating magnetic field generation device is used to produce a dynamic rotating magnetic field, which guides the motion of the arc spot by changing the direction and the strength of the magnetic field, achieving uniform etching of the target surface while reducing the generation of macroparticles and enhancing plasma concentration [98].

Multi-mode magnetic field coupling not only increases target utilization and reduces the ejection of macroparticles significantly but also optimizes the transport path of plasma to improve the uniformity of the deposited coatings and their adhesion to substrates. Moreover, multi-mode magnetic field coupling can be adjusted flexibly according to different process requirements to achieve precise control over arc spot motion and plasma transport, playing an important role in various arc ion plating application scenarios.

The magnetic field configuration designs shown in Figure 9b–d involve coupling an axial focusing guiding magnetic field generation device to a bipolar radial rotating magnetic field generation device. The axial focusing guiding magnetic field generation device consists of an electromagnetic coil wound with enameled wire, with insulation protection inside and outside the coil. It is placed at the front end of the bipolar radial rotating magnetic field generation device and connected to a ring-shaped iron core yoke at the bottom. The coil is powered by direct current, and the strength of the focusing guiding magnetic field is adjusted by varying the current. This axial focusing guiding magnetic field changes the distribution of the magnetic field in the transport space, increases the axial magnetic field strength in the transport space, reduces the plasma constraint on the target surface, and improves the efficiency and density of plasma transport to the transport space [99].

In this multi-mode composite magnetic field configuration design, combining the rotating transverse magnetic field with the axial auxiliary magnetic field and the axial focusing guiding magnetic field to form a multi-magnetic field coupling structure can break through the limitations of traditional single-magnetic field structures and provide various arc spot control magnetic field modes. The bipolar rotating transverse magnetic field is realized through a multi-pole iron core frame and enameled wire winding coils, and is excited by a three-phase variable-frequency sine alternating current power supply with a phase difference of 120°. The magnetic field direction is transverse and rotatable. The axial auxiliary magnetic field is set behind the target and is generated by a direct current electromagnetic coil to assist in controlling the motion of the arc spot and enhancing discharge stability. The focusing guiding magnetic field is set at the flange end and is generated by a direct current electromagnetic coil to focus the plasma and improve transport efficiency. A combination of the bipolar rotating transverse magnetic field generation device, the axial magnetic field generation device, and the coaxial focusing magnetic field yoke can produce various composite magnetic fields. For example, a single-type composite magnetic field is formed by the bipolar rotating transverse magnetic field alone to control the motion of the arc spot; a two-type composite magnetic field is formed by coupling the bipolar rotating transverse magnetic field with the axial magnetic field at the flange end; and gradient or uniform composite magnetic fields are formed by one or multiple axial magnetic field devices. Different magnetic field modes, used either individually or in combination, can not only meet the arc spot magnetic field requirements near the target surface, improve the discharge form, reduce power density, and decrease the ejection of macroparticles, but also optimize the magnetic field distribution in the plasma transport space to enhance the transport efficiency and the uniformity [100].

**Figure 10:**
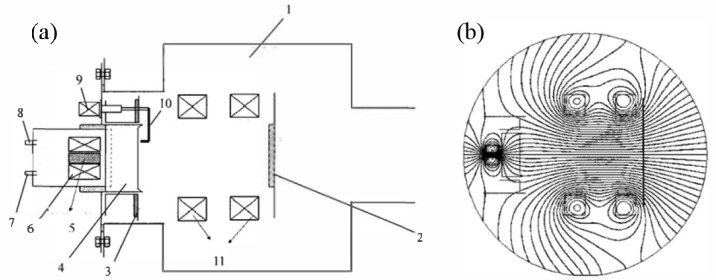
Schematic diagram of the coupled magnetic field arc ion plating device and the corresponding magnetic field line distribution: (**a**) Schematic diagram of the coupled magnetic field deposition device; (**b**) Distribution of magnetic field lines from the target to the substrate inside the vacuum chamber. 1. Vacuum chamber; 2. Substrate; 3. Magnetic ring; 4. Target; 5. Nickel-plated pure iron; 6. Electromagnetic coil; 7. Inlet pipe; 8. Outlet pipe; 9. Arc ignition coil; 10. Arc ignition needle; 11. Coupled enhanced magnetic field coil.

In addition to optimizing the magnetic field design for arc spot control and the plasma transport constraint design of the focusing magnetic field, the coupled magnetic field-assisted arc ion plating deposition device can still be further optimized in terms of plasma transport space distribution. As shown in Figure 10a, the AIP deposition device is equipped with two sets of magnetic field generation devices, which assists deposition through magnetic field coupling, solving the problem of uneven plasma distribution in the transport space in traditional processes. The magnetic field device behind the target is used to control the motion of the arc spot, and two coupled enhanced oscillating magnetic field coils are placed in front of the substrate inside the vacuum chamber. The two coupled enhanced magnetic field coils are coaxial with the target, as shown in Figure 10b. The magnetic field generated by these coils and the magnetic field generated by the device placed behind the target form an S-N-S-N-S-N distribution. The coupled magnetic field of the two coils can better confine the transport of plasma, by reducing plasma loss during transport and improving the deposition rate and uniformity of the deposited coatings. Moreover, the double electromagnetic coils in front of the sample act as a long magnetic filter system, which can reduce macroparticles deposition on the surface and improve the quality of the deposited coatings [101].

## 4. Conclusions and Outlook

### 4.1. Conclusions

This paper reviews the progress on the structure of circular arc sources and magnetic field arc control technology in AIP, with a focus on the design characteristics of different circular arc source structures and the optimization of magnetic field configurations. It is evident that circular arc sources, with their simple structure, convenient installation, flexible target combinations, high cooling efficiency, and high ionization and deposition rates, have shown significant application potential in AIP technology. In terms of magnetic field arc control technology, advancements from traditional single-magnetic-field configurations to multi-level magnetic field coupling, static and dynamic magnetic field coupling, and multi-mode magnetic field coupling have significantly enhanced the controllability of arc spot motion and the efficiency of plasma transport. This, in turn, has improved the uniformity and the adhesion of the coatings, reduced the generation of macroparticles, and optimized the coating quality.

The magnetic field configurations in arc ion plating have evolved from traditional single-field structures to advanced multi-coupling systems, demonstrating a clear trend toward dynamic regulation and composite design. Permanent magnet rotating and axisymmetric configurations lay the foundation for basic arc spot control, while rotating transverse and multi-mode alternating electromagnetic coupling systems enable precise manipulation of arc spot trajectories and plasma distribution. The multi-magnetic field structure coupling, integrating multi-level, static–dynamic, and multi-mode designs, represents the cutting-edge approach to enhancing target utilization, reducing macroparticles ejection, and improving coating uniformity. This technological progression underscores the continuous pursuit of optimizing arc ion plating processes for high-performance coating applications in diverse industrial sectors (Table 2). Multi-level magnetic field coupling technology introduces multiple magnetic field layers with different gradients, designing multiple magnetic field regions around the target to achieve multi-level adjustable arc spot motion patterns. This technology not only enables rapid rotation and uniform distribution of the arc spot by adjusting the strength and direction of the magnetic field but also optimizes the trajectory of the arc spot and the efficiency of plasma transport by dynamically regulating the gradient and the distribution of the magnetic field. Static and dynamic magnetic field coupling technology combines the stability of static magnetic fields with the flexibility of dynamic magnetic fields, achieving precise control over arc spot motion and plasma transport through carefully designed spatiotemporal distributions of the magnetic fields. Multi-mode magnetic field coupling technology further integrates various modes of magnetic fields, combining the characteristics of static and dynamic magnetic fields to achieve comprehensive optimization of arc spot motion and plasma transport, improving target utilization and coating quality significantly.

### 4.2. Outlook

In the field of circular arc sources and their magnetic field arc control technology, although significant research progress has been made, there is still room for improvement. The current research focus is on improving target utilization, reducing the generation of macro-particles, and optimizing the adhesion and wear resistance of coatings. With the continuous improvement of coating performance requirements in various fields, the development of new magnetic field arc control technologies, the exploration of more efficient arc source structural designs, and the realization of intelligent coating process control have become key directions for future research.

The existing technologies have obvious bottlenecks in dynamic regulation systems and composite magnetic field structures. For example, the multi-pole excitation coils of rotating transverse magnetron arc sources need to be matched with specific frequency conversion power supplies, leading to increased system hardware costs and parameter debugging relying on experience, which makes batch process replication difficult. The dynamic superposition of magnetic fields in multi-mode alternating electromagnetic coupling arc sources requires precise matching of current frequencies, but the response delay of existing feedback systems causes the arc spot motion trajectory to lag behind the magnetic field changes. In addition, the magnetic yoke materials of multi-stage magnetic field coupling devices are prone to permeability decay under plasma bombardment, and the hysteresis effect caused by the superposition of static and dynamic magnetic fields will affect the stability of arc spot splitting, thereby influencing coating performance.

Future research can be deeply optimized in multiple aspects. Through the combination of numerical simulation and experiments, the relationship between magnetic field configurations and arc spot motion will be studied in depth, and more advanced magnetic field simulation and optimization algorithms will be developed to achieve more precise magnetic field control. New composite magnetic field structures, such as multi-stage magnetic field coupling and the collaborative effect of dynamic and static magnetic fields, will be explored to improve plasma transmission efficiency and coating uniformity. Combining materials science and artificial intelligence technologies, high-performance coating materials suitable for complex working conditions will be developed to realize intelligent control and real-time monitoring of the arc ion plating process, enhancing the stability and reliability of the coating process.

However, despite the great potential of artificial intelligence technology in intelligent control, there are still some limitations. For example, issues such as sensor accuracy, response time, and system robustness under different operating conditions still need further research and improvement. In addition, the complexity of artificial intelligence algorithms may lead to increased computational costs, which need to be considered in practical industrial applications. Therefore, future research needs to leverage the advantages of artificial intelligence technology while addressing these potential challenges to ensure its effective application in the arc ion plating process.

## Figures and Tables

**Table 1:** Comparison table of arc source structures in arc ion plating.

Arc Source Type	Structural Characteristics	Limitations
Circular Arc Source	Simple structure and easy installation, enabling flexible combination of multiple targets for multi-element coatings	Limited target utilization under traditional fixed magnetic field design Single magnetic field configuration cannot completely avoid macroparticles ejection
Conical Arc Source	Conical structure with complex design	High production and processing costs, with magnetic field interference issues Poor arc control effect and low cooling efficiency
Rectangular Planar Arc Source	Rectangular planar target with a single structure	Single target, difficult to prepare multi-element coatings Low target utilization and high coating cost Prone to forming V-shaped grooves after long-term use
Cylindrical Arc Source	Cylindrical target with fixed installation position	Single installation position, low coating efficiency Poor flexibility in deposition process, small discharge area Requires complex motion devices

**Table 2:** Comparison table of magnetic field configurations in arc ion plating.

Magnetic Field Configuration Type	Structural Characteristics	Core Advantages
Permanent Magnet Rotating Magnetron Arc Source	- Adopts rotatable permanent magnet devices (e.g., eccentric monopolar axial magnetic field, eccentric transverse magnetic field, eccentric arched magnetic field) - Dynamically adjustable magnetic field configurations (e.g., eccentric monopolar magnetic field forming an asymmetric axial distribution on the target surface)	- Dynamically adjusts the arc spot motion area through rotating magnetic fields, improving arc uniformity - Expands the discharge area, reduces power density, and decreases macroparticles ejection - Significantly improves target utilization (e.g., eccentric monopolar magnetic field makes arc spots form a “semicircular” trajectory covering the entire target surface)
Axisymmetric Magnetron Arc Source	- Installs an adjustable current electromagnetic coil behind the target, with a high-permeability pure iron core at the center - Magnetic field shows axisymmetric distribution on the target surface (maximum transverse magnetic field strength at the edge, zero at the center)	- Precisely controls magnetic field strength by adjusting current, enabling accurate regulation of arc spot motion - As magnetic field strengthens, arc spots transform from random motion to stable rotation at the edge, covering the entire target surface - Improves target utilization and coating uniformity
Rotating Transverse Magnetron Arc Source	- Sets up a rotating magnetic field generator around the target (multiple magnetic poles + excitation coils), powered by two-phase/three-phase excitation with phase differences - Magnetic field rotation speed and strength can be adjusted via current frequency and magnitude	- Dynamic rotating magnetic field transforms arc spots from concentrated spots to dispersed arc lines, completely covering the target surface - Reduces macroparticles ejection, achieves distributed discharge, and significantly improves coating quality and uniformity - Strong adjustability of magnetic field parameters to adapt to different process requirements
Multi-Mode Alternating Electromagnetic Coupling Arc Source	- Composed of axisymmetric magnetic field and focusing guidance magnetic field devices - Dynamically coupled magnetic fields (e.g., axisymmetric divergent magnetic field + reverse focusing magnetic field) can change periodically to form arched coupled magnetic fields	- Precisely controls arc spot motion through dynamically superimposed magnetic fields (e.g., pushing arc spots outward or constraining them at the center) - Enhances plasma ionization rate and transmission efficiency, reducing macroparticles ejection - Driven by multi-waveform currents, enabling multiple arc spot control modes
Multi-Magnetic Field Structure Coupling Arc Source	**Multi-Level Magnetic Field Coupling** - Inner coupled magnetic field (behind the target) coordinates with two sets of outer coupled magnetic fields (in front of the target) to form dynamic arched magnetic fields and axial focusing magnetic fields **Static-Dynamic Magnetic Field Coupling** - Combination of permanent magnets (static) and electromagnetic coils (dynamic), e.g., central transverse + edge longitudinal permanent magnets combined with alternating coil currents **Multi-Mode Magnetic Field Coupling** - Integration of axisymmetric, transverse, and rotating magnetic fields, e.g., central magnetic group + secondary transverse rotating magnetic field + axial focusing guidance magnetic field	**Multi-Level Coupling** - Multi-level adjustable arc spot motion modes, optimizing plasma transmission efficiency **Static-Dynamic Coupling** - Combines stability and flexibility, reducing local over-etching of the target and improving ionization rate **Multi-Mode Coupling** - Comprehensive control of arc spot trajectories (spiral/annular motion), enhancing target utilization and coating adhesion

## Data Availability

No new data were created or analyzed in this study. Data sharing is not applicable to this article.
